# Supplementary figures and images for: Hypoglycemic and Antioxidant Properties of Extracts and Fractions from Polygoni Avicularis Herba
Source: Molecules. 2022 May 24;27(11):3381. doi: 10.3390/molecules27113381 (PMC9182118; doi:10.3390/molecules27113381)

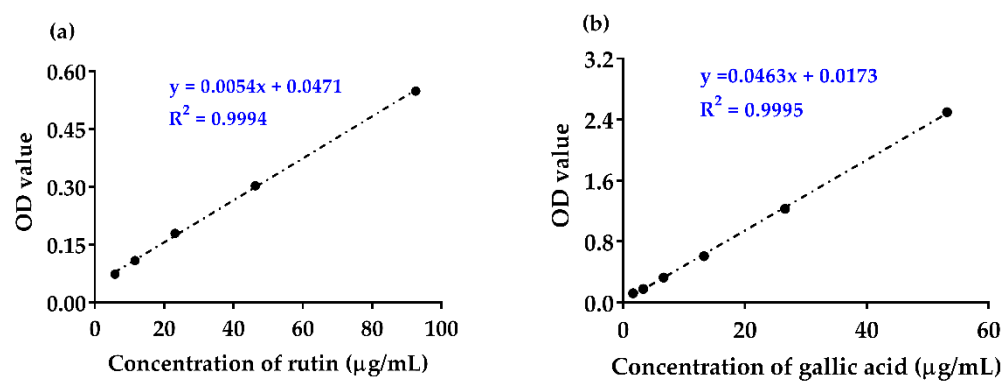

**Figure. S1** Linear fit results of rutin and gallic acid.

Supplement: Supplementary file 1 [file molecules-27-03381-s001.zip › molecules-1684032-supplementary.pdf]
